# Induced Pluripotent Stem Cell-Derived Neural Stem Cell Transplantations Reduced Behavioral Deficits and Ameliorated Neuropathological Changes in YAC128 Mouse Model of Huntington's Disease

**DOI:** 10.3389/fnins.2017.00628

**Published:** 2017-11-10

**Authors:** Abeer Al-Gharaibeh, Rebecca Culver, Andrew N. Stewart, Bhairavi Srinageshwar, Kristin Spelde, Laura Frollo, Nivya Kolli, Darren Story, Leela Paladugu, Sarah Anwar, Andrew Crane, Robert Wyse, Panchanan Maiti, Gary L. Dunbar, Julien Rossignol

**Affiliations:** ^1^Field Neurosciences Institute Laboratory for Restorative Neurology, Central Michigan University, Mount Pleasant, MI, United States; ^2^Program in Neuroscience, Central Michigan University, Mount Pleasant, MI, United States; ^3^Department of Psychology, Central Michigan University, Mount Pleasant, MI, United States; ^4^Field Neurosciences Institute, St. Mary's of Michigan, Saginaw, MI, United States; ^5^College of Medicine, Central Michigan University, Mt Pleasant, MI, United States

**Keywords:** neural stem cells, Huntington's disease, cell transplantations, YAC128, iPSCs, iPS-NSCs

## Abstract

Huntington's disease (HD) is a genetic neurodegenerative disorder characterized by neuronal loss and motor dysfunction. Although there is no effective treatment, stem cell transplantation offers a promising therapeutic strategy, but the safety and efficacy of this approach needs to be optimized. The purpose of this study was to test the potential of intra-striatal transplantation of induced pluripotent stem cell-derived neural stem cells (iPS-NSCs) for treating HD. For this purpose, we developed mouse adenovirus-generated iPSCs, differentiated them into neural stem cells *in vitro*, labeled them with Hoechst, and transplanted them bilaterally into striata of 10-month old wild type (WT) and HD YAC128 mice. We assessed the efficiency of these transplanted iPS-NSCs to reduce motor deficits in YAC128 mice by testing them on an accelerating rotarod task at 1 day prior to transplantation, and then weekly for 10 weeks. Our results showed an amelioration of locomotor deficits in YAC128 mice that received iPS-NSC transplantations. Following testing, the mice were sacrificed, and their brains were analyzed using immunohistochemistry and Western blot (WB). The results from our histological examinations revealed no signs of tumors and evidence that many iPS-NSCs survived and differentiated into region-specific neurons (medium spiny neurons) in both WT and HD mice, as confirmed by co-labeling of Hoechst-labeled transplanted cells with NeuN and DARPP-32. Also, counts of Hoechst-labeled cells revealed that a higher proportion were co-labeled with DARPP-32 and NeuN in HD-, compared to WT- mice, suggesting a dissimilar differentiation pattern in HD mice. Whereas significant decreases were found in counts of NeuN- and DARPP-32-labeled cells, and for neuronal density measures in striata of HD vehicle controls, such decrements were not observed in the iPS-NSCs-transplanted-HD mice. WB analysis showed increase of BDNF and TrkB levels in striata of transplanted HD mice compared to HD vehicle controls. Collectively, our data suggest that iPS-NSCs may provide an effective option for neuronal replacement therapy in HD.

## Introduction

Huntington's disease (HD) is a progressive, neurodegenerative, genetic disorder characterized by choreic movements, behavioral and cognitive disturbances, and dementia (Craufurd et al., 2001). The disease is caused by an autosomal dominant mutation in the *huntingtin* gene (*HTT*), and the mode of inheritance is dominant with almost full penetration (with 40 or more CAG repeats). The genetic basis of HD was discovered in 1993 (MacDonald et al., 1993), and it was found to be caused by an elongated Cystosine-Adenine-Guanine (CAG) repeat on the short arm of chromosome 4p16.3 in the *HTT* gene. HD symptoms include psychiatric, motor, and cognitive deficits, and are variable among patients during early stages of the pathology. However, as the disease progresses, symptoms become predictable, with all patients eventually developing same characteristic pathologies (Walker, 2007). The life expectancy for HD patients ranges between 15 and 20 years after the appearance of motor symptoms (Landles and Bates, 2004; Walker, 2007). The most apparent and earliest damage is seen in the neostriatum, which is composed of the caudate nucleus and putamen (Walker, 2007). Medium spiny neurons (MSNs) in the striatum appear to be the most vulnerable neurons to the damage in HD (Albin et al., 1990).

Different animal models, either chemically or genetically induced, have been developed to study various aspects of HD. One of these models, theYAC128 HD mouse, contains the full-length human mutant *HTT* (m*HTT*) inserted into its genome which results in the expression of m*HTT* with 128 CAG repeats (Slow et al., 2003). YAC128 mice show selective, age-dependent, striatal and cortical atrophy and neurodegeneration, and develop progressive deterioration of motor and cognitive functions (Van Raamsdonk et al., 2005; Gray et al., 2008; Ehrnhoefer et al., 2009). The decline in motor abilities manifests as progressive deficits in accelerating rotarod performance that correlates with the loss of neurons in the striatum (Slow et al., 2003).

Experimental approaches used to treat HD aim to decrease the levels of the mHTT protein, improve the survivability of neurons, as well as to replace the affected neurons. Stem cell therapy holds significant promise for treating HD, and includes the transplantation of stem cells into the affected regions of the brain (Cundiff and Anderson, 2011). Owing to the promising outcomes in animal models of HD, the use of stem cell transplants in human clinical trials have been performed to test for efficacy (Clelland et al., 2008). Transplants of fetal tissue, embryonic stem cells (ESCs), mesenchymal stem cells (MSCs), and neuronal stem cells (NSCs; both adult and differentiated from ESCs) have been used as experimental treatments for HD, and all possess different properties that may be beneficial for use as a therapy. Although some promising results have been found following the transplantation of any of these cell sources, the immune rejection of the graft (Bernreuther et al., 2006; Cicchetti et al., 2014) as well as some serious adverse events have been reported. Specifically, tumor formation has been found following ESC transplants (Aubry et al., 2008), and hemorrhage with multiple solid and cystic lesions in the brain was found following fetal tissue grafting (Keene et al., 2009). While the anti-inflammatory properties and ability for autologous transplantations result in a superior survivability of MSC transplants (Rossignol et al., 2009, 2011; Dey et al., 2010; Lin et al., 2011; Sadan et al., 2012; Serrano Sánchez et al., 2014), these cells do not readily differentiate into neurons (Przyborski et al., 2008), limiting their utility as a source of cellular replacement. Unlike transplants of MSCs, NSC transplants have the potential for replacing neurons that have degenerated within the targeted region (Vazey et al., 2006; Yang and Yu, 2009), yet the survivability of NSCs post-transplantation remains poor with signs of immune rejection (Johann et al., 2007; Rossignol et al., 2014). Similarly, the accessibility to NSCs is limited, and met with technical and ethical complications caused by the demand for using embryonic tissue for isolation.

A new source of pluripotent stem cells emerged when Takahashi and Yamanaka (2006) generated pluripotent stem cells by reprogramming somatic cells through inserting 4 genes (*OCT4, SOX2, Klf4*, and *c-Myc*) into fibroblasts obtained from the skin. These cells, defined as induced pluripotent stem cells (iPSCs), were able to differentiate into any cell type in the body (Takahashi and Yamanaka, 2006). iPSCs appear to have much of the same characteristics as ESCs, including morphology and differentiation capabilities, as well as the capacity to form teratomas containing cells of all three germ lineages when transplanted into severe combined immune deficient mice (Takahashi and Yamanaka, 2006). However, there are genetic and epigenetic differences between them (Robinton and Daley, 2012). The use of iPSCs offers a viable alternative to ESCs and circumvents the issues of availability and ethical concerns surrounding the use of embryos (Verma and Verma, 2011). Work in our laboratory indicated that transplanting rat-derived iPSCs into the striata of rats given 3- nitropropionic acid to model HD, revealed improvements in the motor function, and differentiation of transplanted cells into region-specific neurons in the striatum (Fink et al., 2014a). However, the transplantation of pluripotent stem cells may pose a risk of over-proliferation if the transplants occur without prior pre-engagement down a given germ layer, such as the neuro-ectoderm (Miura et al., 2009). Subsequently, NSCs that were derived from iPSCs obtained from a patient with juvenile onset HD, were transplanted into the striata of YAC128 mice, and showed significant improvement in motor functions (Jeon et al., 2014). This study demonstrated a proof-of-principle that therapeutic efficacy can still be derived following the transplantation of pre-engaged iPSCs, which may reveal to be a safer method of therapy.

Overall, accumulating evidence suggests that stem cell transplantations hold significant promise for use as a treatment for HD. However, there are many technical challenges that need to be addressed before this approach can be safely translated as a treatment in clinical cases of HD. This study aimed to further characterize the use of iPS-NSCs as a treatment for HD by utilizing behavioral, histological, and protein analyses as outcome measures for therapeutic efficacy.

We hypothesized that iPS-NSCs will provide a useful alternative for ESCs and NSCs for HD treatment because these cells can be generated in adequate amounts from somatic cells, and form a more personalized treatment that will promote integration of the transplanted cells, with less risk of immune rejection. Also, as iPS-NSCs are more restricted to the neuronal lineage compared to iPSCs, we believe they will confer less of a risk for unwanted proliferation and tumor formation *in vivo*.

This study tested the efficacy of transplanting of iPS-NSCs as a therapy for HD after intrastriatal transplantation in YAC128 HD mice by (1) assessing their effects on motor function; (2) the survivability and differentiation capabilities 10 weeks after transplantation; and (3) the histological and protein analyses 10 weeks after transplantation.

## Materials and methods

### Cell generation and culture

#### iPSC culture

iPSCs used in this study, were generated and characterized as described in a previously published protocol in our laboratory (Fink et al., 2014b). In brief, iPSCs were generated from fibroblasts that were isolated from tails of adult wild type mice. These fibroblasts were reprogrammed into iPSCs by using two adenoviruses (ADs): one contains *Oct4, Sox2*, and *Klf4* and another contains *c-Myc* which are all considered pluripotent factors. The recombinant ADs were developed in our laboratory and described previously in a published protocol (Fink et al., 2014b). The generated iPSCs were confirmed to express pluripotent markers using immunocytochemistry (ICC) and flow cytometry. The cells were then cryopreserved in freezing media containing 10% dimethyl sulfoxide (DMSO) [Medium is composed of 45% knock out serum and 45% of Dulbecco's Modified Eagles Media (DMEM), and 10% DMSO]. The generated iPSCs were thawed and plated on 0.1% gelatin coat, and cultured in iPSC media [DMEM (Life Technologies, Carlsbad, CA) supplemented with 10% knock-out serum, β-mercaptoethanol (Life Technologies, Carlsbad, CA), 1% 1X non-essential amino acids (NEAA; Life Technologies, Carlsbad, CA), 20 ng/mL basic fibroblast growth factor (bFGF; Life Technologies, Carlsbad, CA), 2 μM L-glutamine (Sigma, St. Louis, MO), 5 mg/mL streptomycin and 5 UI/mL penicillin, and 10 ng/mL leukemia inhibitory factor (LIF; Life Technologies, Carlsbad, CA)]. Cells were passaged by dissociating them in Accutase (Sigma, St. Louis, MO), centrifuging at 250 g for 5 min at 4°C, and plating them on 0.1% gelatin coat.

#### iPS-NSCs generation

**Figure 1:**
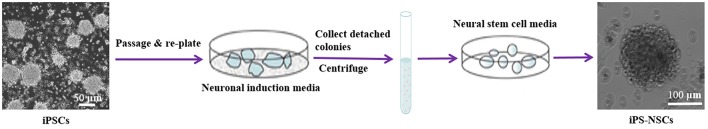
Differentiation of iPSCs into iPS-NSCs. iPSCs were expanded to 80% confluency, and then, the iPSC media were replaced by neuronal induction media (Neurobasal-A, 1X B27-A, 1X N2, 1X NEAA, 1X Glutamax, and 5 mg/mL streptomycin and 5 UI/mL penicillin). The cells were kept in culture until they detached and formed neurospheres. The media containing detached cells were centrifuged, and the pellet was dissociated in Accutase. Cells were then re-plated in neural stem cell media (Neurobasal-A, 1X B27-A, 1X N2, 1X NEAA, 1X Glutamax, 20 ng/mL EGF, 10 ng/mL bFGF, and 5 mg/mL streptomycin and 5 UI/mL penicillin). Cells were passaged three times and then characterized using ICC.

The iPS-NSCs were generated by differentiation of iPSCs following the first stage in a published protocol with some modifications (Niclis et al., 2013). Briefly, iPSCs were expanded to 80% confluency, and then, the iPSC media were replaced by neuronal induction media [Neurobasal-A (Life Technologies, Carlsbad, CA) supplemented with 1X B27-A (Life Technologies, Carlsbad, CA), 1X N2 (Life Technologies, Carlsbad, CA), 1X NEAA (Life Technologies, Carlsbad, CA), 1X Glutamax (Life Technologies, Carlsbad, CA), and 5 mg/mL streptomycin and 5 UI/mL penicillin]. Half of the media was changed every 3 days, and the cells were kept in culture until they detached and formed neurospheres. The media containing detached cells were centrifuged at 100 g for 5 min at 4°C, and the pellet was dissociated in 1 mL Accutase (Sigma, St.louis, Mo) for 5 min at 37°C, then suspended in 5 mL phosphate buffered saline (PBS) and centrifuged another time. Cells were then re-plated in neural stem cell media [Neurobasal-A supplemented with 1X B27-A, 1X N2 (Life technologies, Carlsbad, CA), 1X NEAA, 1X Glutamax (Life Technologies, Carlsbad, CA), 20 ng/mL epidermal growth factor (EGF; Life Technologies, Carlsbad, CA) and 10 ng/mL bFGF (Life Technologies, Carlsbad, CA), and 5 mg/mL streptomycin and 5 UI/mL penicillin] (Figure 1).

#### Characterization of iPS-NSCs

The iPSC-derived neurospheres were passaged every week for 3 weeks and then characterized through ICC for neural lineage specific protein expression (Nestin, Sox2, β-tubulin-III and NeuN). The cells were grown on poly-L-lysine-coated, 25 mm glass coverslips for 2 days, after which cells were washed with PBS (0.01 M at pH 7.4) three times and fixed using 4% paraformaldehyde for 10 min at 4°C. Then a blocking solution (10% normal goat serum in PBS) was added to the coverslips and incubated for 1 h at room temperature. After that, the primary antibodies [Nestin (mouse monoclonal), Sox2 (rabbit polyclonal), and NeuN (rabbit monoclonal); 1:500; Abcam, Cambridge, U.K, β-Tubulin III (chicken polyclonal antibody); 1:300; Aves Labs Inc., Tigard, OR] diluted in PBS containing 0.1% Triton X-100 were added to the assigned wells and incubated at 4°C overnight. The primary antibodies were then aspirated and the coverslips were rinsed 3 times in PBS. Secondary antibodies with either AlexaFluor488 (1:500; goat anti-chicken IgG, or goat anti-rabbit IgG; Invitrogen, Carlsbad, CA), or AlexaFluor594 (1:500; goat anti-mouse IgG; Invitrogen, Carlsbad, CA) were then added and incubated at room temperature for 1 h. After that, the coverslips were rinsed 3 times in PBS. Hoechst-33342 (1:1,000; Sigma, St. Louis, MO) was added to each coverslip for 5 min at room temperature, and then, the coverslips were rinsed 3 times and mounted onto glass slides using Fluoromount reagent (Sigma, St louis, MO). Slides were visualized under fluorescent microscopy (Leica, Germany).

### Animals

All procedures involving animals that were used in this study are approved by the Central Michigan University Institutional Animal Care and Use Committee. Twenty eight, 10-month-old male and female wild type and YAC 128 mice were randomly assigned to groups and housed in cages on a continuous 12-h day/night cycle (from 11:00 to 23:00 h). Mice had access to water and food, *ad libitum*, and they were kept at the same conditions of temperature and humidity.

### Transplantation

#### Preparation of iPS-NSCs for transplantation

On the day of surgery, iPS-NSCs were pre-labeled with 5 μg/mL of Hoechst 33342 (Sigma, St Louis, MO), and re-suspended at a density of 200,000 cells/μL in Hanks' Balanced Salt Solution (HBSS).

#### Surgeries

Surgeries were performed on all mice in the study at 10 months of age. Mice were randomly assigned into one of the following groups (*n* = 7): HD+HBSS, HD+iPS-NSCs, WT+HBSS, and WT+iPS-NSCs. The surgery was conducted under aseptic conditions. Mice were anesthetized using 2.0% isoflurane with 0.8 L/min oxygen maintenance throughout the procedure. The mice were continuously monitored throughout surgery, and adjustments of isoflurane and oxygen supply were made as needed. The back of the head of each mouse was shaved from the line between ears to the frontal part. After that, each anesthetized mouse was placed into the stereotaxic device (Kopf Instruments, Tujunga, CA), and the surgical site of the head was cleaned with chlorhexidine (Molnycke Healthcare, Norcross, GA). Then, a midline incision was made on the scalp, and skin was retracted. Two burr holes were made over the neostriatum (coordinates relative to bregma: anterior +0.5 mm; lateral ± 1.75 mm; with the tooth bar set at −3.3 mm). The iPS-NSCs or HBSS were loaded into a 10 μL Hamilton micro-syringes and every mouse received bilateral injections of cells and/or vehicle at a constant rate of 0.33 μL/min. Each hemisphere was injected with 200,000 cells at 2.5 mm ventral to the dura. After a 3-min rest period, the micro-syringe was moved 0.1 mm dorsally and another 200,000 cells were injected, followed by another 3-min rest period. The syringe was withdrawn slowly and re-positioned over the contralateral hemisphere and the procedure was repeated. Each hemisphere received total of 400,000 cells, while the vehicle control group received 2 μL HBSS. Incisions were closed by using 7-mm sterile wound clips, and analgesic ointment was applied to the incision site. Following surgeries, mice were monitored in recovery cages and transferred to their home-cages when they were fully recovered.

Postoperative care over a 5-day period included monitoring of vital signs, weight, movement, amount of food, water ingested as well as the status of the tissue at the incision site. Intra-peritoneal injections of physiological saline were given for mice showing signs of dehydration during the second post-surgical day. Clips were removed 10 days following the surgery.

### Accelerating rotarod testing

The motor activity of the mice was assessed using the accelerating rotarod (San Diego Instruments; San Diego, CA). Mice were first trained at increasing speed starting from 5 rpm/s and accelerated at 0.5 rpm/s up to 40 rpm on five consecutive trials for 5 days before receiving the treatment. For testing, the mice were placed on the rod, which started rotating at 5 rpm/s and accelerated at 0.5 rpm/s until they fell. Mice were given 5 trials with a 45-s inter-trial interval. Baseline measurements were performed 1 day before the transplantation, and then, testing was done once each week, for 10 weeks after surgery. Motor function was measured by latency to fall (sec) from the accelerating rotarod.

### Histology

#### Immunohistochemistry

Four mice from each group were anesthetized with sodium pentobarbital by intraperitoneal injection, and then, were transcardially perfused first with 0.01 M cold PBS (pH 7.4), followed by 4% paraformaldehyde for fixation of the brains and their brains were extracted and kept in 4% paraformaldehyde for 24 h at 4°C. The brains were then transferred to 30% sucrose in PBS for 48 h at 4°C and then flash-frozen in 2-methylbutane (Sigma, St. Louis, MO) on dry ice for 3 min and stored at −80°C until processing. The brains were sectioned coronally on a cryostat at 40 μm thickness and placed in 6 serial wells.

For immuno-histochemical (IHC) analysis, primary antibodies were used for double labeling of (1) mature neurons (mouse anti-NeuN clone A60, 1:500; Millipore, Billerica, MA) and (2) medium spiny neurons (rabbit monoclonal [EP720Y] to DARPP32, 1:500; Abcam, Cambridge, U.K). Equally spaced sections from each brain was used for labeling of astrocyte reactivity (Rabbit polyclonal to GFAP, 1:500; Abcam, Cambridge, U.K). Tissue sections were blocked using 10% normal goat serum in PBS for 1 h at room temperature, and then transferred to wells containing the primary antibodies in PBS with 0.1% Triton X-100, and incubated at 4°C overnight with continuous agitation. On the following day, the brain sections were rinsed three times in Tris-buffer saline with 0.1% Tween-20 (TBST) and transferred to wells containing the appropriately conjugated secondary antibodies [AlexaFluor488 (goat anti-mouse IgG), or AlexaFluor594 (goat anti-rabbit IgG); 1:1,000); Invitrogen] for 1 h at room temperature. Finally, the sections were rinsed in TBST and mounted onto positively charged glass slides, using Fluoromount media (Sigma, St. Louis, MO).

#### Imaging and analysis

For NeuN and DARPP-32 slides from each mouse, both striatum from 4 randomly selected tissue sections were imaged under fluorescent microscopy, while maintaining a consistent exposure time and fluorescent intensity for each slide. Exposure settings were maintained at 360-, 460-, and 380-ms, respectively, for images obtained from Hoechst, Alexafluor-488, and Alexafluor-594 labeled sections. A 20-μm Z-stack of images was collected from seven individual depths, spaced 3 μm apart. Each striatum was imaged in its entirety by tiling each individual region under a 20-x objective. Following image acquisition, the complete Z-stack from each image was processed using ZEN 2.3 (Carl Zeiss AG; Oberkochen, Germany) through the extended depth of focus to flatten the acquired images, and the tiled image was stitched. Each color channel was exported as individual TIFFfiles and subsequently analyzed, and estimation of total neuronal profiles and area were calculated using MBF Stereo Investigator software (MBF Bioscience; Williston, VT). Counts of DARPP-32 and NeuN labeled cells were done within 200 × 200 counting frames spaced evenly throughout the striatum (grid size was 1,000 × 1,000 mm). Random confocal images were captured using confocal laser microscope (Zeiss; Thornwood, NY) to look at the co-localization of Hoechst labeled cells with DARPP-32 and NeuN. For GFAP, transplants site from each mouse was imaged using a fluorescence microscope (Leica, Germany).

### Western blot

Three brains from each group were used for Western blot (WB) analysis at 54-weeks of age. Mice were sacrificed by cervical dislocation and their brains were extracted and dissected. For every brain, striata were isolated, and lysed in cold radio-immuno-precipitation assay (RIPA) buffer [10 mM Tris-Cl (pH 8.0), 1 mM EDTA, 0.5 mM EGTA, 0.1% SDS, 140 mM NaCl, 0.1% sodium deoxycholate, 1% Triton X-100, with protease inhibitors (Sigma, St. Louis, MO)]. The homogenate was centrifuged at 20 *g* at 4°C for 30 min. The supernatant was taken and aliquoted in PCR tubes and stored at −80°C until use. Protein concentrations for each sample were determined using the Pierce BCA protein assay (Thermo Scientific, Rockford, IL). Samples were mixed with equal amount of 2X SDS-sample buffer (125 mM Tris-HCl, pH 6.8, 4% sodium dodecyl sulfate, 20% glycerol, 10% 2-mercaptoethanol and 0.2% bromophenol blue) and boiled for 2 min. For assessment, equal amount of protein of each sample was loaded and separated on gradient gel (4–20% SDS-PAGE). The SDS-PAGE was run at 100 V with running buffer (25 mM Tris-Base, 192 mM glycine, 0.1% SDS, and 1 mM EDTA). The proteins from gel were transferred overnight to the PVDF membrane (Millipore, Billerica, MA) in an ice cold buffer containing 25 mM Tris–Base, 192 mM glycine and 10% methanol. Following transfer, the blots were rinsed three times in TBST, and the membranes were blocked with 5% fat-free milk in TBST for 1 h. Then, the blots were incubated with primary antibody rabbit anti-BDNF (1:1,000; Sigma, St. Louis, MO) or rabbit anti-TrkB (1:1,000; Cell Signaling Technology, Danvers, MA), and rabbit anti- β-tubulin, (1:1,000; Abcam) in 5% fat-free milk powder dissolved in TBST for overnight at 4°C. Membranes were then rinsed three times with TBST, and incubated with the respective horse radish peroxidase (HRP) conjugated secondary antibodies (goat anti-rabbit IgG; diluted 1:10,000) in 1.5% fat-free milk powder in TBST for 1 h. The membranes were then washed three times with TBST. The blots were then developed with Immobilon^TM^ Western Chemiluminescent HRP-substrate (Millipore, Billerica, MA), and scanned. The optical density of each lane of the blot was measured using ImageJ software (NIH, Bethesda, MD).

### Statistics

All statistical analyses were performed using SPSS v24. Accelerating rotarod data was analyzed using repeated measures analysis of variance (ANOVA). Fall latency, including the baseline and 10 weeks following transplantation, was used for statistical comparisons between wild type and YAC128 mice treated with iPS-NSCs, or received HBSS. One way ANOVA was performed to analyze differences of weekly fall latency amongst all groups. Histological and WB data were analyzed using one way ANOVA. Tukey's Honest Significant Difference (HSD) *post-hoc* test, was performed when the omnibus *F*-values were significant. The alpha level is set at *p* ≤ 0.05 for all analyses.

## Results

### iPS-NSC characterization

**Figure 2:**
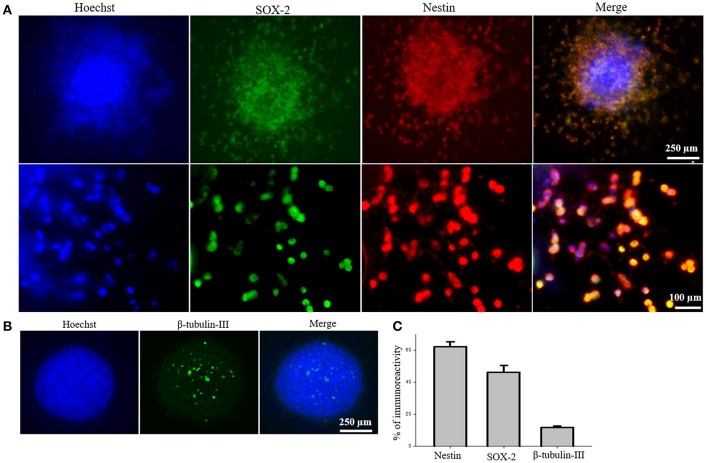
Characterization of iPS-NSCs through ICC. **(A)** Hoechst-labeled iPS-NSCs (blue) showed positive expression of neural stem cells markers; SOX2 (green) and Nestin (red). Upper row shows a neurosphere, and lower row shows individual cells. **(B)** Few cells in the neurosphere showed positive expression of immature neuronal marker, β-tubulin-III (green). **(C)** Percentage of cells expressing Nestin (*M* = 61.9%, *SD* = 14.3), SOX2 (*M* = 46.1%, *SD* = 18.7), and β-tubulin-III (*M* = 11.8%, *SD* = 2.9).

To test the efficacy of iPS-NSC transplantations, we generated iPSCs from WT mice and differentiated them to NSCs. After differentiation of iPSCs to NSCs, ICC was performed to characterize the cells to confirm the expression of neural stem markers. Results of characterization confirmed that neurospheres showed positive expression of SOX2 and Nestin (*M* = 46.1%, *SD* = 18.7; *M* = 61.9%, *SD* = 14.3, respectively; Figures 2A,C). Similarly, cells from the neurospheres showed positive expression of the immature neuronal marker β-Tubulin-III (*M* = 11.8%, *SD* = 2.9; Figures 2B,C), confirming the immature status of neuronal cells derived from the iPS-NSCs.

### Accelerating rotarod

**Figure 3:**
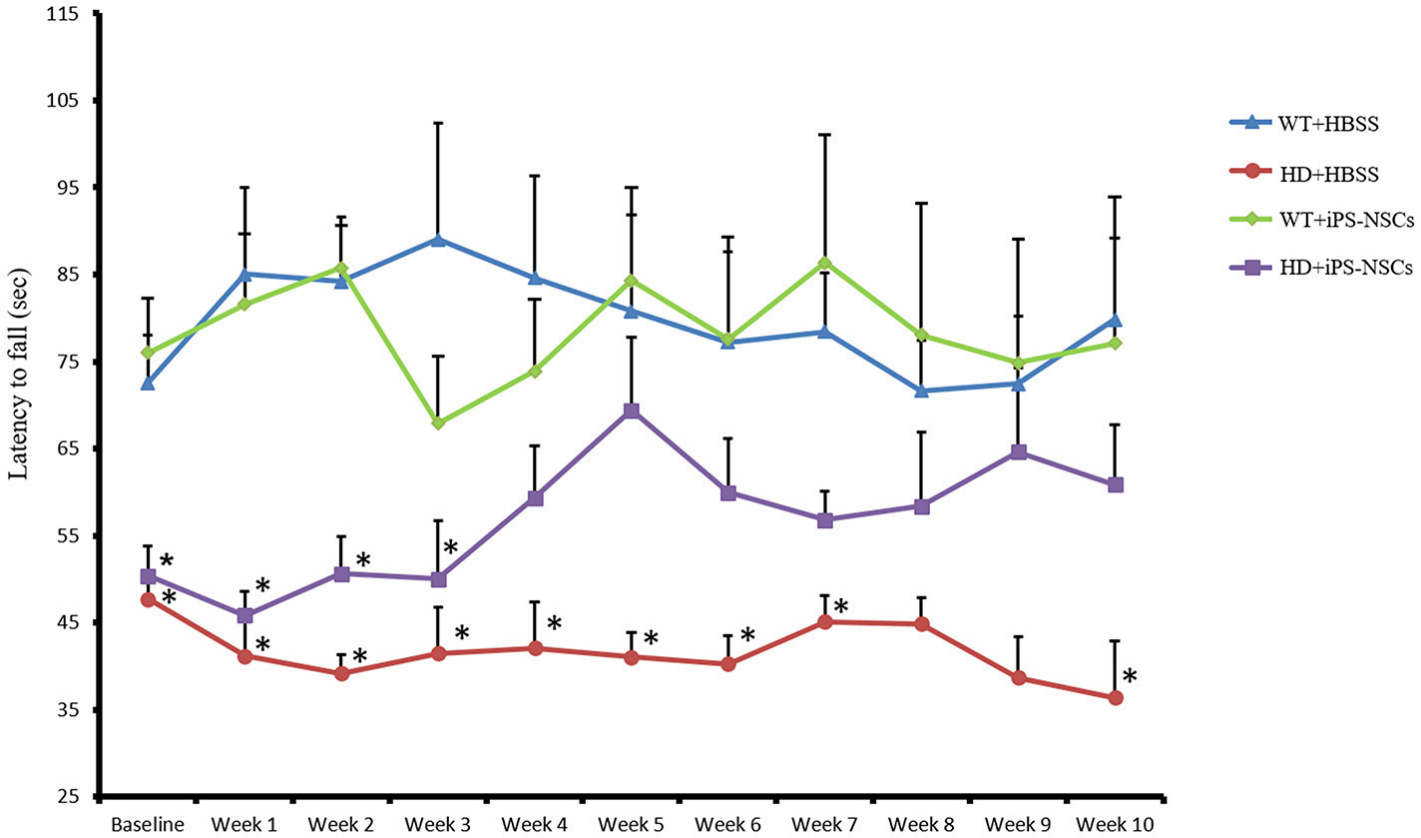
Accelerating rotarod testing. Accelerod testing showed a significant decrease of the fall latency in HD vehicle control compared to WT mice (*p* = 0.001) but no significant decrease in the iPS-NSCs treated HD mice were found compared to WT mice (*p* = 0.077). At the same time, there was no overall significant difference between HD vehicle controls and iPS-NSCs treated HD mice (*p* = 0.336). Weekly testing on the accelerod revealed a significant difference between iPS-NSCs treated HD mice and WT mice at baseline (*p* = 0.017). However, iPS-NSCs treated HD mice showed no significant difference from WT mice starting from Week 4 after transplantation (*p* = 0.159) and up to 10 weeks after transplantation (*p* = 0.605). Also, there was no significant difference between iPS-NSCs treated HD mice and HD vehicle controls (*p* = 0.393) suggesting an intermediate treatment effect at this time point. ^*^significant different from WT+HBSS, *p* < 0.05.

To determine if the transplantation of iPS-NSCs could improve motor function in YAC128 mice, we tested motor coordination using the accelerating rotarod. Repeated-measures ANOVA of accelerating rotarod (accelerod) data demonstrated a significant between-group effect in the performance of the mice over the course of the study [*F*_(3, 24)_ = 8.461, *p* = 0.001]. Tukey *post-hoc* revealed a significant between-group difference in performance on the accelerod, with WT groups demonstrating longer latencies to fall than mice in the HD vehicle control group (*p* = 0.001), thus confirming the motor deficits of this animal model. No significant differences were found between WT group and iPS-NSCs-treated HD mice (*p* = 0.077), nor between HD vehicle-control and the iPS-NSCs-treated HD mice group (*p* = 0.336) (Figure 3 and Tables S1, S2).

One-way ANOVA of weekly accelerod data showed a significant difference at baseline between WT and iPS-NSCs- treated HD mice (*p* = 0.017), but by week 4 after transplantation, the performance of iPS-NSCs-treated HD mice was not significantly different from that of the WT groups (*p* = 0.158). At 10 weeks following transplantation, the HD iPS-NSCs treated HD group was still performing similarly to WT mice (*p* = 0.605), however there was not a significant difference between their performance and that of the HD control animals (*p* = 0.393) suggesting an intermediate treatment effect at this time point. (Table S2).

### Histological results

#### Survivability and differentiation of transplanted iPS-NSCs

**Figure 4:**
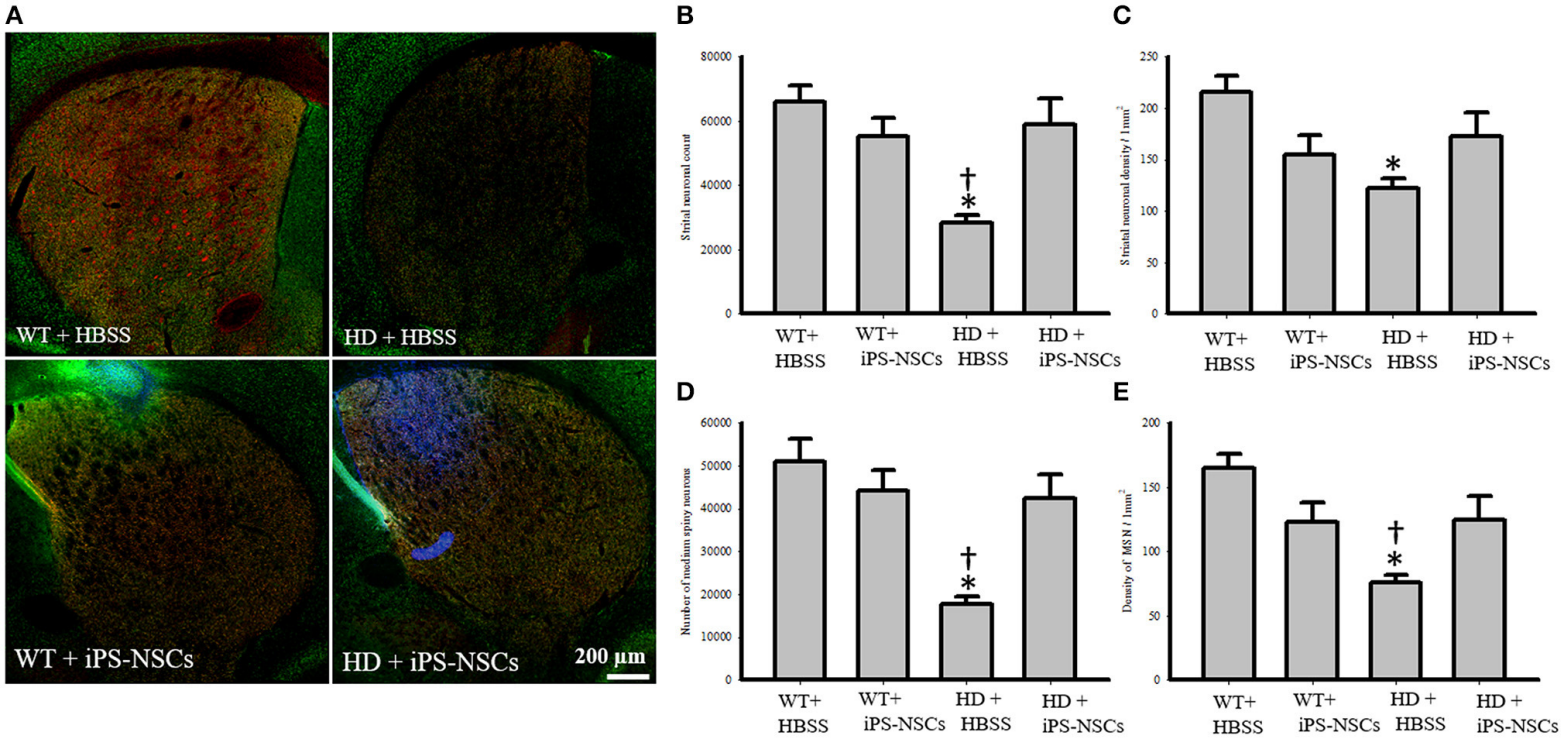
Striatal neuronal and medium spiny neurons counts and densities. **(A)** Stitched images for striatum in each group showing markers of iPS-NSCs (Hoechst labeled cells; blue), mature neurons (NeuN; green) and medium spiny neurons (DARPP-32, red). **(B,D)** Analysis of NeuN and DARPP-32 labeled cells (mature neurons and medium spiny neurons, respectively) in striata showed that HD+HBSS mice are significantly different from WT+HBSS mice (*p* = 0.002), but HD+iPS-NSCs are not significantly different from WT+HBSS mice (NeuN, *p* = 0.79; DARPP-32, *p* = 0.57). Moreover, HD+iPS-NSCs mice are significantly different from HD+HBSS (*p* = 0.01; *p* = 0.01). **(C,E)** Analysis of NeuN and DARPP-32 labeled cells densities (mature neurons and medium spiny neurons, respectively) in striata showed that HD+HBSS mice are significantly different from WT+HBSS mice (*p* = 0.01, *p* = 0.003, respectively), but HD+iPS-NSCs are not significantly different from WT mice (*p* > 0.05). Also, DARPP-32 labeled cells densities were significantly increased in iPS-NSCs-HD treated mice compared to HD vehicle controls (*p* = 0.047). ^*^Significant different from WT+HBSS,^†^significant different from HD+iPS-NSCs, *p* < 0.05.

Hoechst-labeled cells were found in both WT and HD brains 10 weeks post-transplantation. However, fewer transplanted cells (Hoechst-labeled cells) in iPS-NSCs-treated WT mice were observed in the striatum compared to iPS-NSCs-treated HD mice (Figure 4A).

**Figure 5:**
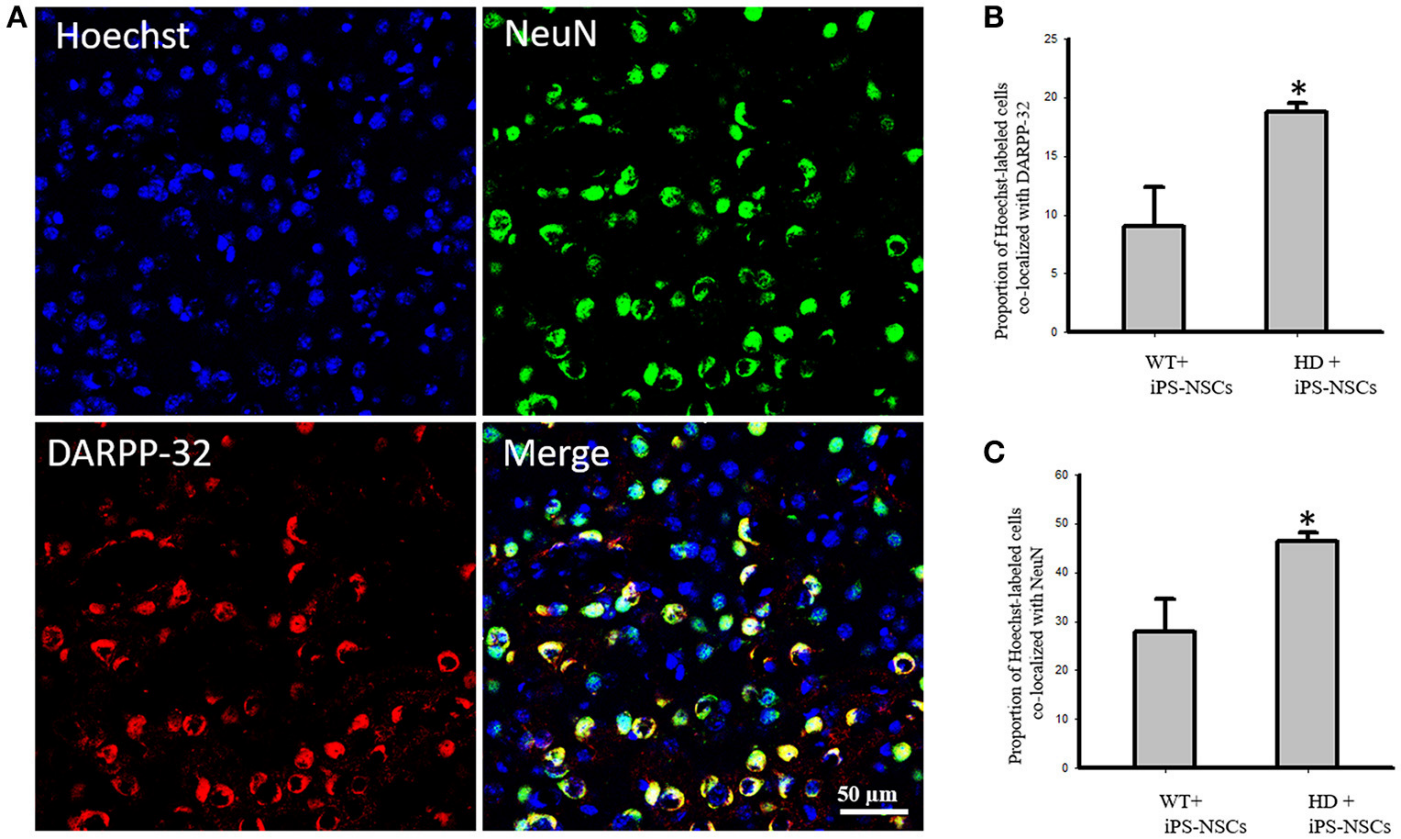
**(A)** Transplanted iPS-NSCs survived and differentiated into mature neurons and medium spiny neurons in HD mice. Confocal images were captured from iPS-NSCs treated HD mouse. Hoechst labeled iPS-NSCs (blue) were found in striata 10 weeks post transplantation. Also, Hoechst labeled iPS-NSCs (blue) show co-expression of mature neurons marker (NeuN; green) and region specific neurons (DARPP-32; red). **(B)** Proportion of Hoechst labeled iPS-NSCs that are co-labeled with DARPP-32 is significantly higher in HD+iPS-NSCs mice compared to WT+iPS-NSCs (*p* = 0.029). **(C)** Proportion of Hoechst labeled iPS-NSCs that are co-labeled with NeuN is significantly higher in HD+iPS-NSCs mice compared to WT+iPS-NSCs (*p* = 0.038). ^*^Significant different, *p* < 0.05.

We observed that Hoechst-labeled cells co-localized with NeuN and DARPP-32 in both WT and HD mice. However, a higher proportion of Hoechst-labeled cells were co-labeled with DARPP-32 and NeuN in HD- (*M* = 18.8%, *SD* = 1.52; *M* = 46.3%, *SD* = 3.56, respectively) compared to WT-mice (*M* = 9.00%, *SD* = 6.63; *M* = 27.88%, *SD* = 13.39, respectively; Figures 5A–C).

Analysis of the stereologically acquired cell-count data revealed significant between-group differences in DARPP-32 and NeuN labeled cells in the striatum [*F*_(3, 12)_ = 9.512, *p* = 0.002, *F*_(3, 12)_ = 8.573, *p* = 0.003, respectively]. Specifically, Tukey *post-hoc* analysis showed that there was a significant difference between WT and HD vehicle control groups in DARPP-32 and NeuN stained cells (*p* = 0.002). However, there was no significant difference between WT mice and the iPS-NSCs-treated HD group (*p* = 0.573 for DARPP-32, *p* = 0.793 for NeuN). Furthermore, there was a significant difference between HD vehicle control and iPS-NSCs-treated HD mice (*p* = 0.014 for DARPP-32, *p* = 0.011 for NeuN; Figures 4A,B,D).

In addition, results of stereologically acquired density measures revealed significant between-group differences in the density of NeuN and DARPP-32 in striata [*F*_(3, 12)_ = 5.031, *p* = 0.017, *F*_(3, 12)_ = 7.392, *p* = 0.001]. Tukey *post-hoc* analysis revealed a significant reduction in NeuN- and DARPP-32 labeled cell densities in HD vehicle-control mice in comparison to the WT mice (*p* = 0.012, *p* = 0.002, respectively), but these differences were not seen between iPS-NSCs-treated HD and WT control groups (*p* = 0.35, *p* = 0.29, respectively), Moreover, the density of the DARPP-32 labeled cells was significantly elevated in iPS-NSCs-HD treated mice compared to HD vehicle controls (*p* = 0.047; Figures 4A,C,E).

#### Astrocyte response at the transplant site

**Figure 6:**
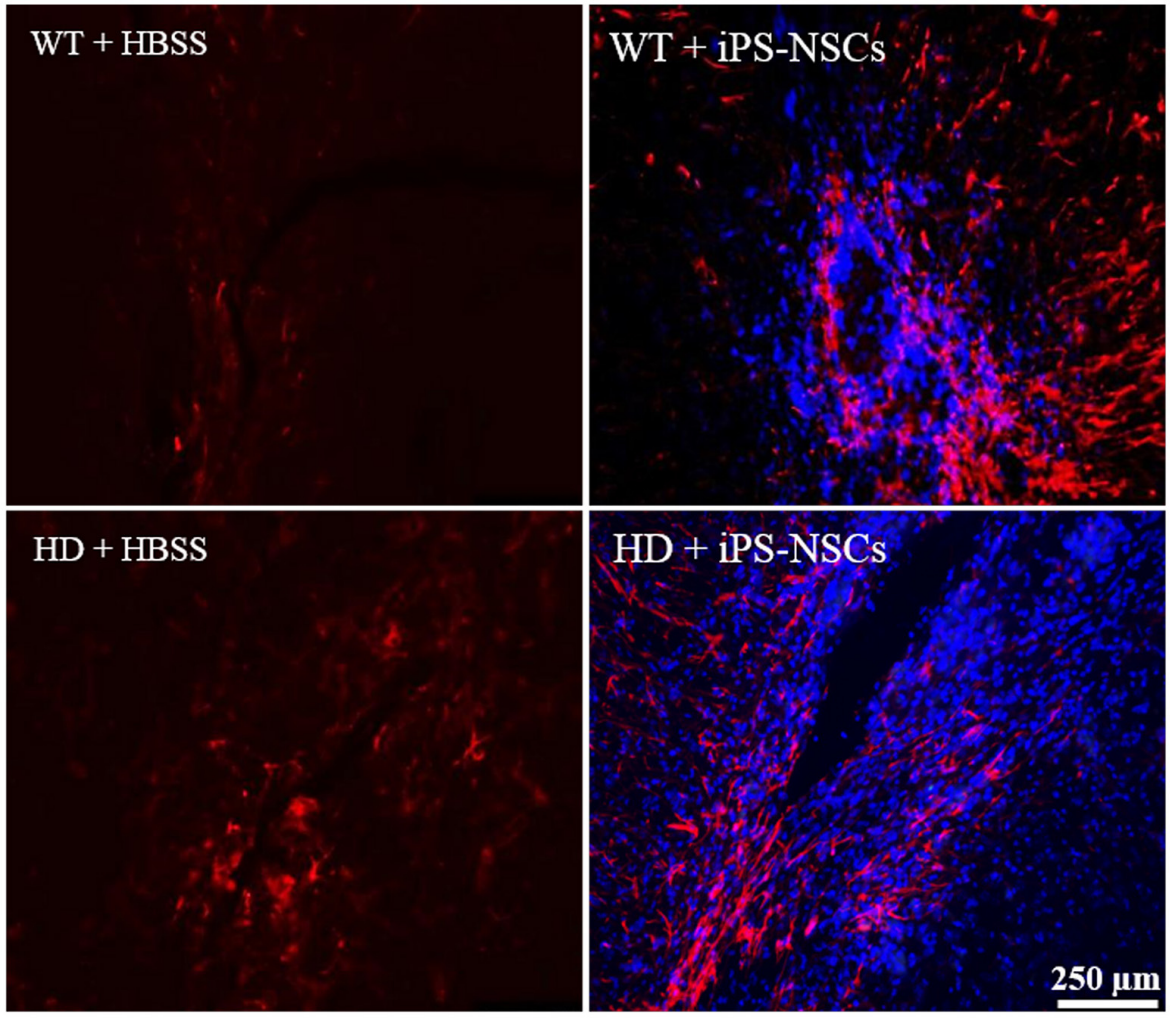
Astrocyte response at the injection site following transplantation. Greater activation of astrocytes (GFAP; red) was observed in iPS-NSCs (Hoechst labeled cells; blue) transplanted brains in comparison to vehicle controls and in iPS-NSCs transplanted WT mice in comparison to iPS-NSCs transplanted HD mice.

Reactive astrocytes were observed at the transplantation site. Higher astrocytic (GFAP; red) responses were observed in iPS-NSCs (Hoechst labeled cells; blue) transplanted brains in comparison to vehicle controls. Also, higher astrocytes response was observed in iPS-NSCs-transplanted WT mice in comparison to iPS-NSCs-transplanted HD mice (Figure 6).

### Western blotting of striatal BDNF and TrkB

**Figure 7:**
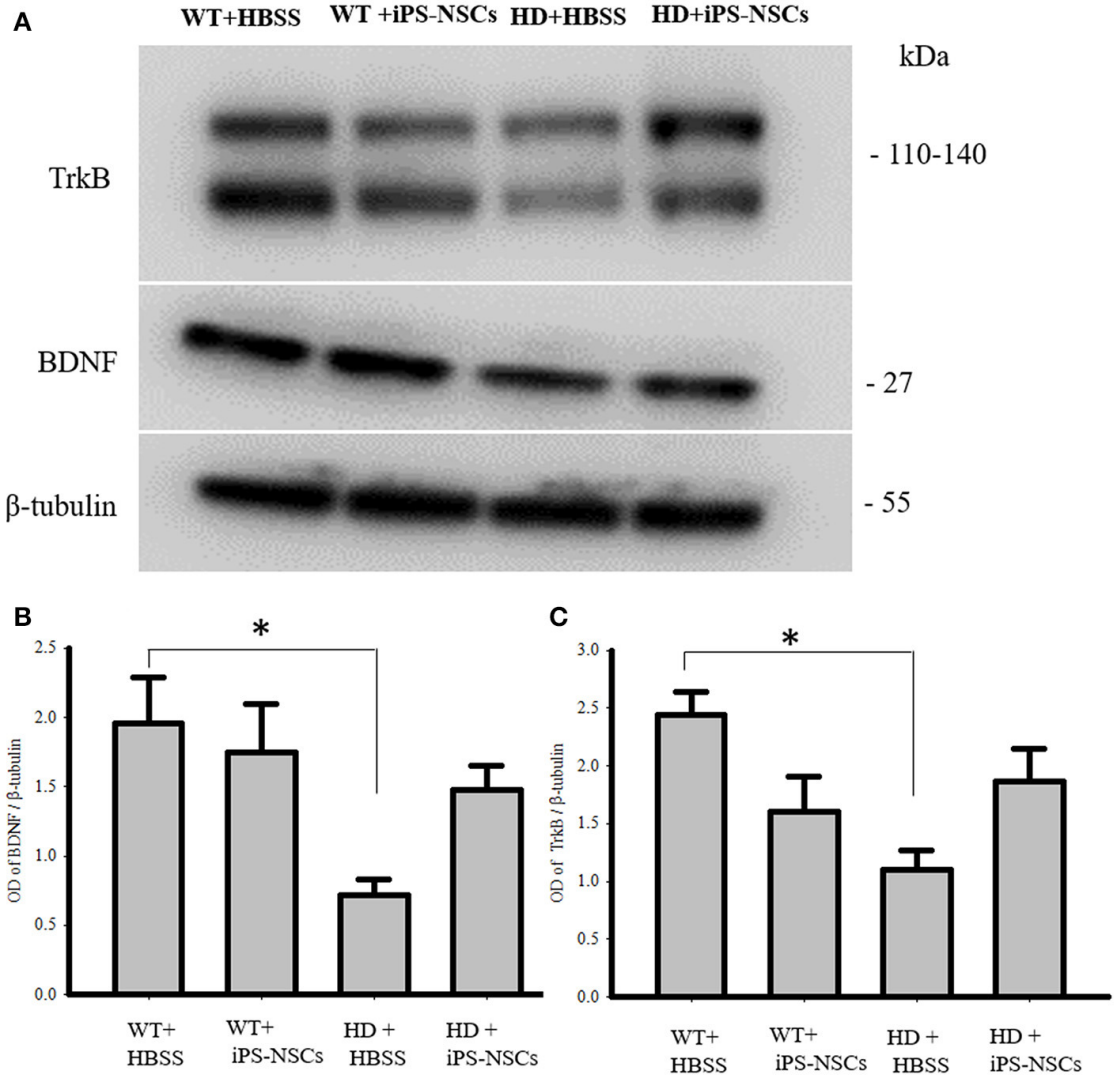
Western blot analysis of BDNF and TrkB. **(A,B)** Western blot analysis showed a significant decrease of BDNF in striata of HD+HBSS compared to WT+HBSS mice (*p* = 0.04), but HD+iPS-NSCs were not significantly different from WT mice (*p* = 0.59), and HD+HBSS mice (*p* = 0.25) suggesting intermediate treatment effect. **(A,C)** Western blot analysis showed a significant decrease of total TrkB in striata of HD+HBSS compared to WT+HBSS mice (*p* = 0.02), but HD+iPS-NSCs were not significantly different from WT+HBSS mice (*p* = 0.31), and HD+HBSS (*p* = 0.31) suggesting intermediate treatment effect. ^*^Significant different from WT+HBSS, *p* < 0.05.

Analysis of WB data showed that there were significant between-group differences in BDNF and TrkB levels in striata [*F*_(3, 8)_ = 4.250, *p* = 0.045 & *F*_(3, 8)_ = 4.739, *p* = 0.035, respectively]. Tukey *post-hoc* analysis showed that there was a significant reduction in BDNF and TrkB in HD vehicle control mice compared to WT mice (*p* = 0.041, *p* = 0.02, respectively). However, there was no significant difference between iPS-NSCs-treated HD mice and WT group (*p* = 0.59, *p* = 0.31, respectively; Figure 7). Although there was no significant difference between iPS-NSCs treated HD mice and HD vehicle controls in levels of BDNF (*p* = 0.24), and TrkB (*p* = 0.31), levels were also not significantly different in iPS-NSCs-treated HD mice when compared to WT mice, suggesting an intermediate treatment effect.

## Discussion

The histological and motor deficits that are seen in the YAC128 mouse model recapitulates the HD changes in human HD patients, which makes this model ideal for assessing therapeutic interventions (Slow et al., 2003). The onset of motor symptoms in HD patients correlates with the striatal neuronal degeneration, and typically appear before the onset of neurodegeneration (Vonsattel et al., 1985). Similarly, in the YAC128 model, motor deficits on the accelerod start at 6 months of age before the onset of neuronal loss, and progress to hypokinesis by 12 months of age. Neuronal degeneration is seen in the striata as early as 9 months of age, with significant neuronal loss developing by 12 months of age (Van Raamsdonk et al., 2005). In this study, we transplanted iPS-NSCs into 10 months old mice and followed them for 10 weeks after transplantation. This age of YAC128 mice is equivalent to the middle age in human (Flurkey et al., 2007), which corresponds with the time of the evident motor deficits in HD patients, and pathological changes in HD brains (Walker, 2007). The defined progressive neuronal degeneration that is accompanied with a deterioration in accelerating rotarod performance in YAC128 mice is crucial in predicting the phenotype which helps in assessing therapeutic interventions.

The primary findings of the present study were that: (1) iPS-NSC transplants improved motor abilities in HD-treated mice; (2) iPS-NSCs survived for at least 10 weeks after transplantation in both WT and HD mice brains; (3) iPS-NSCs differentiated into mature neurons and region-specific neurons (medium spiny neurons); (4) increases in protein levels of BDNF and TrkB were found in iPS-NSCs-treated HD mice; and (5) differentiation patterns of the transplanted iPS-NSCs were dissimilar between HD and WT mice.

Consistent with previous studies in YAC128 mice, this study found significant motor deficits in the performance of HD mice on the accelerating rotarod, as evidenced by a decreased latency to fall relative to WT mice. Decreases in motor abilities, such as those necessary for performance on the accelerod, are related to striatal dysfunction that is usually preceded by neuronal loss (Van Raamsdonk et al., 2005). This study utilized iPS-NSCs as a treatment to ameliorate these motor deficits, and indicated that HD mice that received iPS-NSC transplantation showed behavioral sparing of motor performance on the accelerod. Additionally, significantly more neurons were found within the striatum of treated YAC128 mice compared to HD vehicle controls. Prior to our study, Jeon et al. (2014) transplanted iPSC-derived neuronal precursors generated from an HD patient into YAC128 mice, and found a significant increase in the latency to fall during the accelerating rotarod compared to HD vehicle controls. However, iPSCs generated from HD patients were found to show the HD phenotypes both *in vitro* (Hd iPSC Consortium, 2012) and *in vivo* (Jeon et al., 2012). In this study, we transplanted NSCs derived from iPSCs that were generated from WT mice. Although the transplantations in our study were allogenic, the use of immunosuppressants could help integrate the transplanted cells in host tissue more efficiently, and lead to even better outcomes in motor performance.

In iPS-NSCs-treated HD mice, Hoechst-labeled cells were found co-localized with DARPP-32 and NeuN within the striatum, which suggests a regional specificity to the patterns of differentiation *in vivo*. The results from this study found a significant reduction of both total neurons, as well as medium-spiny neurons, in HD vehicle controls mice at 54 weeks of age. This is consistent with previous findings and confirms the progressive neuronal degeneration attributed to HD (Slow et al., 2003). However, in HD mice treated with iPS-NSCs, more neurons, including more medium-spiny neurons were found within the striatum, with a significant portion of these co-labeling with Hoechst. Although both the higher neuronal count, and the appropriate regional-specific pattern of differentiation could be detected following the transplantation of iPS-NSCs into HD mice, it is difficult to determine whether the transplanted cells were able to functionally integrate into the neuronal architecture without further investigations using methods in electrophysiology. Therapeutic benefits from cell transplantations can be derived from both cellular replacement, such as neuronal differentiation and integration, or by the release of trophic factors such as BDNF that can support the survival of neurons in compromised neurodegenerative conditions such as HD (Dey et al., 2010; Serrano Sánchez et al., 2014; Pollock et al., 2016). In this study, it may also be possible that the transplantation of these iPS-NSCs functioned to protect endogenous neurons from further degeneration by releasing neurotropic factors such as BDNF.

The role of BDNF in promoting neuronal survival and function has been demonstrated across several neuropathological conditions including HD (Strand et al., 2007; Zuccato and Cattaneo, 2007). In HD, BDNF and the BDNF receptors (TrkB) are substantially reduced as a consequence of epigenetic and transcriptional regulation (Zuccato et al., 2008), and BDNF-TrkB signaling is thought to be a major contributor to the progressive neuronal degeneration (Ginés et al., 2006). Medium-spiny neurons are a particularly sensitive population of neurons to BDNF dysfunction due to the necessity of maintaining its elaborate architecture (Zuccato and Cattaneo, 2007). This study also found a deficit in BDNF levels in HD mice, but following the transplantation of iPS-NSCs, a trend toward increased BDNF was found in striata of HD mice. It is possible that this increase in BDNF could be derived either from the transplanted cells, from preservation of endogenous neurons, or most likely a combination of transplant-derived BDNF that helped preserve endogenous neurons, leading to additional endogenous-derived BDNF. In either event, the increase of BDNF within the striatum of iPS-NSCs treated HD mice supports the utility of this treatment to improve the microenvironment of the HD brain. We also found a trend toward increased total TrkB levels in striata of iPS-NSCs transplanted HD mice compared to HD vehicle controls. There are two major TrkB isoforms; the full-length and the truncated isoforms (Fryer et al., 1996). The full length isoform contains an intracellular tyrosine kinase domain and it is activated by BDNF, while the truncated form lacks the intracellular kinase activity and contains a unique C-terminal (Eide et al., 1996). The function of truncated TrkB receptors is still not well described, but it was reported that they act as modulators of BDNF responsiveness; they sequester and translocate BDNF, and induce cascades of intracellular signaling. In addition, they play a role in enhancing neurite growth and modifying cytoskeletal structures (Carim-Todd et al., 2009; Fenner, 2012). The ultimate effectiveness of BDNF depends on the distribution and expression of TrkB receptors including the full-length and truncated isoforms (Fenner, 2012). The increase in total TrkB receptors in iPS-NSCs treated HD mice compared to HD vehicle controls in this study could be as a result from the increases in neurons, as well as BDNF levels that are found in straita of transplanted HD mice. This increase in total TrkB levels may have played a role in modulating BDNF-TrkB signaling and led to preservation of endogenous neurons.

With regards to the migration of iPS-NSCs post-transplantation, a dissimilar pattern of migration was observed between WT and HD mice. In HD-treated mice, Hoechst-labeled cells were found predominately within the striatum. However in WT-iPS-NSCs-treated mice, Hoechst-labeled cells were found predominately surrounding the needle track, with some labeled cells identified within the corpus callosum and cortex. This represents an interesting finding that suggests a differential pattern of migration of these cells post-transplantation in the HD condition. NSCs such as iPS-NSCs are known to express chemokine receptors such as CXCR4 (Stewart et al., 2017). The progressive neurodegeneration found in HD results in a substantially increased inflammatory response, which is accompanied by reactive microglia as well as increased levels of cytokines and chemokines (Träger et al., 2015). An increase in chemokines within the sriata of HD mice may function to facilitate the distribution of the transplanted iPS-NSCs within the striatum. WT mice on the other hand, lack this inflammatory response and could reasonably promote a less specific distribution of these cells throughout the brain, with fewer total cells enticed to leave the initial site. Similar to promoting migration of transplanted iPS-NSCs, an increase in chemokines and cytokines in HD mice may also encourage altered patterns of differentiation and survival, which could explain why a greater number of transplanted iPS-NSCs could be found in HD mice compared to WT mice in this study.

Although not explicitly quantified in this study, an increase in reactive astrogliosis was observed surrounding the needle track in iPS-NSCs treated WT compared to iPS-NSCs HD treated mice. The reason for this discrepancy is unknown, however, several factors might account for this. First, it may be a function of an altered and dysfunctional capacity for glial scar formation in HD compared to WT mice. This could be due to co-morbid pathologies existing in HD, such as a weakened blood-brain barrier (Drouin-Ouellet et al., 2015). The increase in reactive gliosis may also be a function of transplanted iPS-NSCs differentiating more into astrocytes in WT mice compared to HD mice, with more integration surrounding the immediate transplant site. If HD mice possess a decreased ability for astrocyte function, then this may have important implications for the role of astrocyte dysfunction in supporting and protecting neurons in the HD condition (Maragakis and Rothstein, 2006). In either regard, further characterization of both the role of inflammation in transplant survival, differentiation, and migration, as well as the disturbance of astrocyte function in HD needs to be further studied.

Of important note, as iPSCs are generated through viral incorporation of oncogenes, there are concerns of insertion and subsequent mutagenesis, or of a permanent expression of the oncogenes, that could transform the transplanted cells into tumors (Rossignol et al., 2014). No tumors were found in this study, which is likely a result of pre-engaging the cells to a committed neuro-ectodermal lineage. The lack of tumors found in this study supports the approach of pre-differentiating of pluripotent cell lines prior to transplantation, however, the risk of having heterogeneous population containing some pluripotent stem cells within the transplanted cells is still possible (Miura et al., 2009). The need for optimizing the differentiation protocols, and sorting cells before transplantations to avoid undifferentiated cells is critical for the safety when translating iPS-NSCs for clinical applications. Also, many studies have shown that induced neural stem cells (iNSCs) can be generated directly by reprogramming somatic cells and bypassing the pluripotent stem cell stage (Lujan et al., 2012; Yu et al., 2015). This could also offer an alternative means of obtaining NSCs with less risk of tumor formation (Hemmer et al., 2014). However, since the protocols for direct differentiation of somatic human cells into NSCs use exogenous genes, tumor formation is still a concern when using induced cells for transplantations (Choi et al., 2017). Whether adopting the iPS-NSCs or iNSCs approach, optimizing of protocols for reprogramming cells is needed to reduce the risk of tumor formation, and to provide a safe and effective treatment with clinical utility.

The current study provides an additional step in a long line of research supporting the clinical relevance of iPS-NSC transplantation in HD. We observed survival of transplanted iPS-NSCs up to 10-weeks following transplantation, with differentiation of transplanted cells into region specific neurons, and a behavioral sparing of motor performance. Collectively, these findings support the hypothesis that iPS-NSCs may prove to be a viable cell-replacement strategy with a high potential for therapeutic benefit. In order to fully assess the utility of transplantation of iPS-NSCs for cell replacement therapies in HD, future work will focus on long-term transplantation while analyzing behavioral function in HD mice.

## Author contributions

AA-G: conception and design, collection and/or assembly of data, data analysis and interpretation, manuscript writing. RC: participated in the design of the study, collection and/or assembly of data. AS: collection and/or assembly of data, manuscript writing. BS, KS, LF, NK, DS, LP, and SA: collection and/ or assembly of data. AC and RW: generation of iPSCs. PM: design, administrative support, data analysis and interpretation, manuscript writing. GD and JR: conception and design, administrative support, data analysis and interpretation, manuscript writing, final approval of manuscript.

### Conflict of interest statement

The authors declare that the research was conducted in the absence of any commercial or financial relationships that could be construed as a potential conflict of interest.

## Abbreviations

ANOVA: analysis of variance
BCA: bicinchoninic acid assay
BDNF: brain derived neurotrophic factor
bFGF: basic fibroblast growth factor
DARPP32: Dopamine- and cAMP-regulated phosphoprotein of 32 kDa
DMEM: Dulbecco's Modified Eagles medium
EDTA: Ethylene-di-amino-tetra-acetic-acid
EGF: epidermal growth factor
GFAP: glial fibrillary acidic protein
HBSS: Hank's balanced salt solution
HD: Huntington's disease
HRP: Horseradish peroxidase
iNSC: induced neural stem cells
iPSC: Induced pluripotent stem cells
iPS-NSCs: induced pluripotent stem cells-derived neural stem cells
Klf4: Kruppel-like factor 4
MSN: Medium spiny neuron
NEAA: non-essential amino acids
NeuN: Neuronal nuclei
NSC: Neural stem cell
OCT4: Octamer-binding transcription factor 4
OD: Optical density
PBS: Phosphate buffer saline
PFA: Paraformaldehyde
PVDF: Polyvinylidene fluoride
QA: Quinolinic acid
RIPA: Radio immunoprecipitation assay
SDS: Sodium dodecyl sulfate
SDS-PAGE: sodium dodecyl sulfate polyacrylamide gel electrophoresis
SOX2: SRY (sex determining region Y)-box 2
TBS: Tris buffer saline
TrkB: Tropomyosin-related kinase B
YAC: Yeast artificial chromosome.

## References

[B1] Albin R. L. Young A. B. Penney J. B. Handelin B. Balfour R. Anderson K. D.. (1990). Abnormalities of striatal projection neurons and N-methyl-D-aspartate receptors in presymptomatic Huntington's disease. New Engl. J. Med. 322, 1293–1298. 10.1056/NEJM199005033221807 1691447

[B2] Aubry L. Bugi A. Lefort N. Rousseau F. Peschanski M. Perrier A. L. (2008). Striatal progenitors derived from human ES cells mature into DARPP32 neurons *in vitro* and in quinolinic acid-lesioned rats. Proc. Natl. Acad. Sci. U.S.A. 105, 16707–16712. 10.1073/pnas.0808488105 18922775 PMC2575484

[B3] Bernreuther C. Dihné M. Johann V. Schiefer J. Cui Y. Hargus G.. (2006). Neural cell adhesion molecule L1-transfected embryonic stem cells promote functional recovery after excitotoxic lesion of the mouse striatum. J. Neurosci. 26, 11532–11539. 10.1523/JNEUROSCI.2688-06.2006 17093074 PMC6674779

[B4] Carim-Todd L. Bath K. G. Fulgenzi G. Yanpallewar S. Jing D. Barrick C. A.. (2009). Endogenous truncated TrkB. T1 receptor regulates neuronal complexity and TrkB kinase receptor function *in vivo*. J. Neurosci. 29, 678–685. 10.1523/JNEUROSCI.5060-08.2009 19158294 PMC2719435

[B5] Choi K. A. Choi Y. Hong S. (2017). Stem cell transplantation for Huntington's diseases. Methods. [Epub ahead of print]. 10.1016/j.ymeth.2017.08.017 28867501

[B6] Cicchetti F. Lacroix S. Cisbani G. Vallières N. Saint-Pierre M. St-Amour I.. (2014). Mutant huntingtin is present in neuronal grafts in huntington disease patients. Ann. Neurol. 76, 31–42. 10.1002/ana.24174 24798518

[B7] Clelland C. D. Barker R. A. Watts C. (2008). Cell therapy in Huntington disease. Neurosurg. Focus 24, E9. 10.3171/FOC/2008/24/3-4/E8 18341412

[B8] Craufurd D. Thompson J. C. Snowden J. S. (2001). Behavioral changes in Huntington disease. Cogn. Behav. Neurol. Behav. Neurol. 14, 219–226. 11725215

[B9] Cundiff P. E. Anderson S. A. (2011). Impact of induced pluripotent stem cells on the study of central nervous system disease. Curr. Opin. Genet. Dev. 21, 354–361. 10.1016/j.gde.2011.01.008 21277194 PMC3932563

[B10] Dey N. D. Bombard M. C. Roland B. P. Davidson S. Lu M. Rossignol J.. (2010). Genetically engineered mesenchymal stem cells reduce behavioral deficits in the YAC 128 mouse model of Huntington's disease. Behav. Brain Res. 214, 193–200. 10.1016/j.bbr.2010.05.023 20493905

[B11] Drouin-Ouellet J. Sawiak S. J. Cisbani G. Lagacé M. Kuan W. L. Saint-Pierre M.. (2015). Cerebrovascular and blood–brain barrier impairments in Huntington's disease: potential implications for its pathophysiology. Ann. Neurol. 78, 160–177. 10.1002/ana.24406 25866151

[B12] Ehrnhoefer D. E. Butland S. L. Pouladi M. A. Hayden M. R. (2009). Mouse models of Huntington disease: variations on a theme. Dis. Models Mech. 2, 123–129. 10.1242/dmm.002451 19259385 PMC2650190

[B13] Eide F. F. Vining E. R. Eide B. L. Zang K. Wang X. Y. Reichardt L. F. (1996). Naturally occurring truncated trkB receptors have dominant inhibitory effects on brain-derived neurotrophic factor signaling. J. Neurosci. 16, 3123–3129. 8627351 10.1523/JNEUROSCI.16-10-03123.1996 PMC2710135

[B14] Fenner B. M. (2012). Truncated TrkB: beyond a dominant negative receptor. Cytokine Growth Factor Rev. 23, 15–24. 10.1016/j.cytogfr.2012.01.002 22341689

[B15] Fink K. D. Crane A. T. Lévêque X. Dues D. J. Huffman L. D. Moore A. C.. (2014a). Intrastriatal transplantation of adenovirus-generated induced pluripotent stem cells for treating neuropathological and functional deficits in a rodent model of Huntington's disease. Stem Cells Transl. Med. 3, 620–631. 10.5966/sctm.2013-0151 24657963 PMC4006485

[B16] Fink K. D. Rossignol J. Lu M. Lévêque X. Hulse T. D. Crane A. T.. (2014b). Survival and differentiation of adenovirus-generated induced pluripotent stem cells transplanted into the rat striatum. Cell Transplant 23, 1407–1423. 10.3727/096368913X670958 23879897

[B17] Flurkey K. Currer J. M. Harrison D. E. (2007). The mouse in aging research, in The Mouse in Biomedical Research, 2nd Edn., eds Fox J. G. Davisson M. T. Quimby F. W. Barthold S. W. Newcomer C. E. Smith A. L. (Burlington, MA: American College Laboratory Animal Medicine; Elsevier), 637–672.

[B18] Fryer R. H. Kaplan D. R. Feinstein S. C. Radeke M. J. Grayson D. R. Kromer L. F. (1996). Developmental and mature expression of full-length and truncated TrkB, receptors in the rat forebrain. J. Comp. Neurol. 374, 21–40. 10.1002/(SICI)1096-9861(19961007)374:1<21::AID-CNE2>3.0.CO;2-P 8891944

[B19] Ginés S. Bosch M. Marco S. Gavalda N. Díaz-Hernández M. Lucas J. J.. (2006). Reduced expression of the TrkB receptor in Huntington's disease mouse models and in human brain. Eur. J. Neurosci. 23, 649–658. 10.1111/j.1460-9568.2006.04590.x 16487146

[B20] Gray M. Shirasaki D. I. Cepeda C. André V. M. Wilburn B. Lu X. H.. (2008). Full-length human mutant huntingtin with a stable polyglutamine repeat can elicit progressive and selective neuropathogenesis in BACHD mice. J. Neurosci. 28, 6182–6195. 10.1523/JNEUROSCI.0857-08.2008 18550760 PMC2630800

[B21] Hd iPSC Consortium (2012). Induced pluripotent stem cells from patients with Huntington's disease show CAG-repeat-expansion-associated phenotypes. Cell Stem Cell 11, 264–278. 10.1016/j.stem.2012.04.027 22748968 PMC3804072

[B22] Hemmer K. Zhang M. van Wüllen T. Sakalem M. Tapia N. Baumuratov A.. (2014). Induced neural stem cells achieve long-term survival and functional integration in the adult mouse brain. Stem Cell Rep. 3, 423–431. 10.1016/j.stemcr.2014.06.017 25241741 PMC4265999

[B23] Jeon I. Choi C. Lee N. Im W. Kim M. Oh S. H.. (2014). *In vivo* roles of a patient-derived induced pluripotent stem cell line (HD72-iPSC) in the YAC128 model of Huntington's disease. Int. J. Stem Cells 7, 43. 10.15283/ijsc.2014.7.1.43 24921027 PMC4049731

[B24] Jeon I. Lee N. Li J. Y. Park I. H. Park K. S. Moon J.. (2012). Neuronal Properties, *in vivo* effects, and pathology of a Huntington's disease patient-derived induced pluripotent stem cells. Stem Cells 30, 2054–2062. 10.1002/stem.1135 22628015

[B25] Johann V. Schiefer J. Sass C. Mey J. Brook G. Krüttgen A.. (2007). Time of transplantation and cell preparation determine neural stem cell survival in a mouse model of Huntington's disease. Exp. Brain Res. 177, 458–470. 10.1007/s00221-006-0689-y 17013619

[B26] Keene C. D. Chang R. C. Leverenz J. B. Kopyov O. Perlman S. Hevner R. F.. (2009). A patient with Huntington's disease and long-surviving fetal neural transplants that developed mass lesions. Acta Neuropathol. 117, 329–338. 10.1007/s00401-008-0465-0 19057918 PMC2676786

[B27] Landles C. Bates G. P. (2004). Huntingtin and the molecular pathogenesis of Huntington's disease. EMBO Rep. 5, 958–963. 10.1038/sj.embor.7400250 15459747 PMC1299150

[B28] Lin Y. T. Chern Y. Shen C. K. Wen H. L. Chang Y. C. Li H.. (2011). Human mesenchymal stem cells prolong survival and ameliorate motor deficit through trophic support in Huntington's disease mouse models. PLoS ONE 6:e22924. 10.1371/journal.pone.0022924 21850243 PMC3151281

[B29] Lujan E. Chanda S. Ahlenius H. Südhof T. C. Wernig M. (2012). Direct conversion of mouse fibroblasts to self-renewing, tripotent neural precursor cells. Proc. Natl. Acad. Sci. U.S.A. 109, 2527–2532. 10.1073/pnas.1121003109 22308465 PMC3289376

[B30] MacDonald M. E. Ambrose C. M. Duyao M. P. Myers R. H. Lin C. Srinidhi L. (1993). A novel gene containing a trinucleotide repeat that is expanded and unstable on Huntington's disease chromosomes. Cell 72, 971–983. 10.1016/0092-8674(93)90585-E 8458085

[B31] Maragakis N. J. Rothstein J. D. (2006). Mechanisms of disease: astrocytes in neurodegenerative disease. Nat. Rev. Neurol. 2, 679. 10.1038/ncpneuro0355 17117171

[B32] Miura K. Okada Y. Aoi T. Okada A. Takahashi K. Okita K.. (2009). Variation in the safety of induced pluripotent stem cell lines. Nat. Biotechnol. 27, 743–745. 10.1038/nbt.1554 19590502

[B33] Niclis J. C. Pinar A. Haynes J. M. Alsanie W. Jenny R. Dottori M.. (2013). Characterization of forebrain neurons derived from late-onset Huntington's disease human embryonic stem cell lines. Front. Cell. Neurosci. 7:37. 10.3389/fncel.2013.00037 23576953 PMC3617399

[B34] Pollock K. Dahlenburg H. Nelson H. Fink K. D. Cary W. Hendrix K.. (2016). Human mesenchymal stem cells genetically engineered to overexpress brain-derived neurotrophic factor improve outcomes in Huntington's disease mouse models. Mol. Ther. 24, 965–977. 10.1038/mt.2016.12 26765769 PMC4881765

[B35] Przyborski S. A. Maltman D. J. Hardy S. A. (2008). Mesenchymal stem cells as mediators of neural differentiation. Curr. Stem Cell Res. Ther. 3, 43–52. 10.2174/157488808783489471 18220922

[B36] Robinton D. A. Daley G. Q. (2012). The promise of induced pluripotent stem cells in research and therapy. Nature 481, 295–305. 10.1038/nature10761 22258608 PMC3652331

[B37] Rossignol J. Boyer C. Lévèque X. Fink K. D. Thinard R. Blanchard F.. (2011). Mesenchymal stem cell transplantation and DMEM administration in a 3NP rat model of Huntington's disease: morphological and behavioral outcomes. Behav. Brain Res. 217, 369–378. 10.1016/j.bbr.2010.11.006 21070819

[B38] Rossignol J. Boyer C. Thinard R. Remy S. Dugast A. S. Dubayle D. (2009). Mesenchymal stem cells induce a weak immune response in the rat striatum after allo or xenotransplantation. J. Cell. Mol. Med. 13, 2547–2558. 10.1111/j.1582-4934.2008.00657.x 20141619 PMC9181362

[B39] Rossignol J. Fink K. Davis K. Clerc S. Crane A. Matchynski J.. (2014). Transplants of adult mesenchymal and neural stem cells provide neuroprotection and behavioral sparing in a transgenic rat model of Huntington's disease. Stem Cells 32, 500–509. 10.1002/stem.1508 23939879

[B40] Sadan O. Shemesh N. Barzilay R. Dadon-Nahum M. Blumenfeld-Katzir T. Assaf Y.. (2012). Mesenchymal stem cells induced to secrete neurotrophic factors attenuate quinolinic acid toxicity: a potential therapy for Huntington's disease. Exp. Neurol. 234, 417–427. 10.1016/j.expneurol.2011.12.045 22285250

[B41] Serrano Sánchez T. Alberti Amador E. Lorigados Pedre L. Blanco Lezcano L. Diaz Armesto I. Bergado J. A. (2014). BDNF in quinolinic acid lesioned rats after bone marrow cells transplant. Neurosci. Lett. 559, 147–151. 10.1016/j.neulet.2013.11.060 24321407

[B42] Slow E. J. Van Raamsdonk J. Rogers D. Coleman S. H. Graham R. K. Deng Y.. (2003). Selective striatal neuronal loss in a YAC128 mouse model of Huntington disease. Hum. Mol. Genet. 12, 1555–1567. 10.1093/hmg/ddg169 12812983

[B43] Stewart A. N. Kendziorski G. Deak Z. M. Brown D. J. Fini M. N. Copely K. L.. (2017). Co-transplantation of mesenchymal and neural stem cells and overexpressing stromal-derived factor-1 for treating spinal cord injury. Brain Res. 1672, 91–105. 10.1016/j.brainres.2017.07.005 28734802

[B44] Strand A. D. Baquet Z. C. Aragaki A. K. Holmans P. Yang L. Cleren C.. (2007). Expression profiling of Huntington's disease models suggests that brain-derived neurotrophic factor depletion plays a major role in striatal degeneration. J. Neurosci. 27, 11758–11768. 10.1523/JNEUROSCI.2461-07.2007 17959817 PMC6673215

[B45] Takahashi K. Yamanaka S. (2006). Induction of pluripotent stem cells from mouse embryonic and adult fibroblast cultures by defined factors. Cell 126, 663–676. 10.1016/j.cell.2006.07.024 16904174

[B46] Träger U. Andre R. Magnusson-Lind A. Miller J. R. Connolly C. Weiss A.. (2015). Characterisation of immune cell function in fragment and full-length Huntington's disease mouse models. Neurobiol. Dis. 73, 388–398. 10.1016/j.nbd.2014.10.012 25447230 PMC4262574

[B47] Van Raamsdonk J. M. Murphy Z. Slow E. J. Leavitt B. R. Hayden M. R. (2005). Selective degeneration and nuclear localization of mutant huntingtin in the YAC128 mouse model of Huntington disease. Hum. Mol. Genet. 14, 3823–3835. 10.1093/hmg/ddi407 16278236

[B48] Vazey E. M. Chen K. Hughes S. M. Connor B. (2006). Transplanted adult neural progenitor cells survive, differentiate and reduce motor function impairment in a rodent model of Huntington's disease. Exp. Neurol. 199, 384–396. 10.1016/j.expneurol.2006.01.034 16626705

[B49] Verma A. Verma N. (2011). Induced pluripotent stem cells and promises of neuroregenerative medicine. Neurol. India 59, 555. 10.4103/0028-3886.84337 21891933

[B50] Vonsattel J. P. Myers R. H. Stevens T. J. Ferrante R. J. Bird E. D. Richardson E. P. (1985). Neuropathological classification of Huntington's disease. J. Neuropathol. Exp. Neurol. 44, 559–577. 10.1097/00005072-198511000-00003 2932539

[B51] Walker F. O. (2007). Huntington's disease. Lancet 369, 218–228. 10.1016/S0140-6736(07)60111-1 17240289

[B52] Yang C. R. Yu R. K. (2009). Intracerebral transplantation of neural stem cells combined with trehalose ingestion alleviates pathology in a mouse model of Huntington's disease. J. Neurosci. Res. 87, 26–33. 10.1002/jnr.21817 18683244 PMC3895944

[B53] Yu K. R. Shin J. H. Kim J. J. Koog M. G. Lee J. Y. Choi S. W. (2015). Rapid and efficient direct conversion of human adult somatic cells into neural stem cells by HMGA2/let-7b. Cell Rep. 10, 441–452. 10.1016/j.celrep.2014.12.038 25600877

[B54] Zuccato C. Cattaneo E. (2007). Role of brain-derived neurotrophic factor in Huntington's disease. Progr. Neurobiol. 81, 294–330. 10.1016/j.pneurobio.2007.01.003 17379385

[B55] Zuccato C. Marullo M. Conforti P. MacDonald M. E. Tartari M. Cattaneo E. (2008). Systematic assessment of BDNF and its receptor levels in human cortices affected by Huntington's disease. Brain Pathol. 18, 225–238. 10.1111/j.1750-3639.2007.00111.x 18093249 PMC8095509

